# Correction: ‘A unified theory for the energy cost of legged locomotion’ (2016), by Pontzer

**DOI:** 10.1098/rsbl.2023.0492

**Published:** 2023-12-13

**Authors:** Herman Pontzer

**Affiliations:** ^1^ Department of Anthropology, Hunter College, 695 Park Avenue, New York, NY 10065, USA; ^2^ New York Consortium for Evolutionary Primatology, New York, NY, USA

**Keywords:** biomechanics, energetics, locomotion

*Biol. Lett.* **12**: 20150935 (Published online 1 February 2016). (https://doi.org/10.1098/rsbl.2015.0935)

Owing to a typographical error that was not caught in the page proofs, equation (3.3) is written incorrectly. The correct equation is

$$E_{\mathrm{COT}}=8\;M^{\text{–}0.34}+50[1+\sin(2\theta \;\text{–}\;74)]\;M^{\text{–}0.12}.$$

This equation will recreate figure 2*c* using the data in electronic supplementary material, table S1. Note that all angular values in the paper, including *θ*, are in degrees.

None of these errors affect the analyses or results presented in the paper.

This has been corrected on the publisher's website.

